# Molecular Diagnosis of Fragile X Syndrome in Subjects with Intellectual Disability of Unknown Origin: Implications of Its Prevalence in Regional Pakistan

**DOI:** 10.1371/journal.pone.0122213

**Published:** 2015-04-14

**Authors:** Madiha Kanwal, Saadia Alyas, Muhammad Afzal, Atika Mansoor, Rashda Abbasi, Flora Tassone, Sajid Malik, Kehkashan Mazhar

**Affiliations:** 1 Human Genetics Program, Department of Animal Sciences, Quaid-i-Azam University Islamabad, 45320 Islamabad, Pakistan; 2 Institute of Biomedical and Genetic Engineering, Islamabad, Pakistan; 3 M.I.N.D. Institute, University of California Davis, Davis, California, United States of America; 4 Department of Biochemistry and Molecular Medicine, University of California Davis, Davis, California, United States of America; TNO, NETHERLANDS

## Abstract

Fragile-X syndrome (FXS) is the most common form of inherited intellectual disability (ID) and affects 0.7–3.0% of intellectually compromised population of unknown etiology worldwide. It is mostly caused by repeat expansion mutations in the *FMR1* at chromosome Xq27.3. The present study aimed to develop molecular diagnostic tools for a better detection of FXS, to assess implementation of diagnostic protocols in a developing country and to estimate the prevalence of FXS in a cohort of intellectually disabled subjects from Pakistan. From a large pool of individuals with below normal IQ range, 395 subjects with intellectual disability of unknown etiology belonging to different regions of the country were recruited. Conventional-PCR, modified-PCR and Southern blot analysis methods were employed for the detection of CGG repeat polymorphisms in the *FMR1* gene. Initial screening with conventional-PCR identified 13 suspected patients. Subsequent investigations through modified PCR and Southern blot analyses confirmed the presence of the *FMR1* mutation, suggesting a prevalence of 3.5% and 2.8% (mean 3.3%) among the male and female ID patients, respectively. These diagnostic methods were further customized with the in-house conditions to offer robust screening of referral patients/families for diagnostics and genetic counseling. Prescreening and early diagnosis are crucial for designing a prudent strategy for the management of subjects with ID. Outcome of the study recommends health practitioners for implementation of molecular based FXS diagnosis in routine clinical practice to give a better care for patients similar to the ones included in the study.

## Introduction

Intellectual disability (ID) is characterized by significantly lower than average intellectual performance and poor adaptive skills that limit one or more daily life activities. It has an onset of symptoms that appear during the early life years and is classified as either ‘mild’, ‘moderate’, ‘severe’ or ‘profound’. It is a serious health problem that render significant social, psychological and economic burden particularly on low income societies. The prevalence of ID has been shown to be higher among males than females, especially measured among children under 15 years of age [1][2]. Reported estimate of the prevalence in Pakistan is 19/1,000 children for severe ID and 65/1,000 children for mild ID [3]. These estimates are considerably higher than in some industrial or developing countries [1].

Fragile X syndrome (FXS, OMIM 300624, X-linked semi-dominant) is the most common cause of ID with a global prevalence of nearly 0.2/1,000 in males and 0.1/1,000 in females. The premutation carrier prevalence is estimated to be 1-3/1,000 in males and 4-7/1,000 in females [4][5]. The phenotype involves a large spectrum of symptoms including ID of various grades, developmental and speech delay, physical characteristics such as large ears, long and narrow jaw, connective tissue problems including hyperextensible metacarpophalangeal (MP) joints, pes plenus and macro-orchidism [6]. The behavioral symptoms of FXS comprise excessive shyness, aggression, hand flapping, hand biting, poor eye contact, tactile defensiveness, anxiety, attention deficit and hyperactivity [7]. Due to the X-linked semi-dominant inheritance pattern, the affected males exhibit a more severe phenotype than the affected females [8].

FXS is mostly caused by the CGG repeat expansion mutations in the *FMR1* (OMIM 309550) at chromosome Xq27.3 which spans 38 kb and contains 17 exons [9]. The protein (FMRP) is an RNA-binding protein, a translational repressor of mRNA targets and plays an important role in synaptic plasticity [10].

The CGG repeat region is highly polymorphic and may comprise from 5–54 units (mean length = 30 repeats). Affected individuals with a full mutation are characterized by a CGG expansion beyond 200 CGG repeats. The expansion of CGG repeats causes the instability of *FMR1* leading to its silencing, absence of FMRP and ultimately to FXS [11–13]. Expansions between 55–200 CGG repeats, called premutations, are generally unmethylated and are unstable during meiosis. These repeats can expand to a full mutation in maternal segregation. The risk of expansion to a full mutation also relies on the size of the premutation [14]. Recent studies have shown the additional contribution of AGG interruptions within the *FMR1* allele [15,16]. In the case of a premutation, the presence of exaggerated amounts of *FMR1* mRNA has been observed in carriers, which leads to a toxic RNA gain-of-function effect and subsequent clinical involvement [17]. Only some premutation carriers manifest significant clinical problems during early life, whereas symptoms like macro-orchidism; cognitive problems and developmental delay; psychiatric problems such as depression and anxiety; hypertension; migraine; sleep apnea; seizure; immune mediated problems including hypothyroidism and fibromyalgia; Fragile-X associated primary ovarian insufficiency and perhaps most importantly a late onset neurological disorder- FXTAS (FX-associated tremor/ataxia syndrome) are observed later in life [18].

The gold standard for molecular detection of FXS diagnosis is PCR analysis coupled with Southern blot. PCR is commonly employed to determine the length of the triplet repeats particularly for the normal/premutation allele while Southern blot can assess the methylation status of the expansion allele. Recently a number of PCR-based methods have been developed for diagnosis of FXS and demonstrated the capability to also amplify *FMR1* alleles within the full mutation range [19][20].

In this study, we have utilized a modified-PCR (bisulfite treatment) based approach to establish the prevalence of FXS in subjects with intellectual disability of unknown origin in various regions of Pakistan. We have customized this method to offer robust FXS diagnostics for subjects with ID of unknown origin.

## Methods

### Subjects

**Fig 1:**
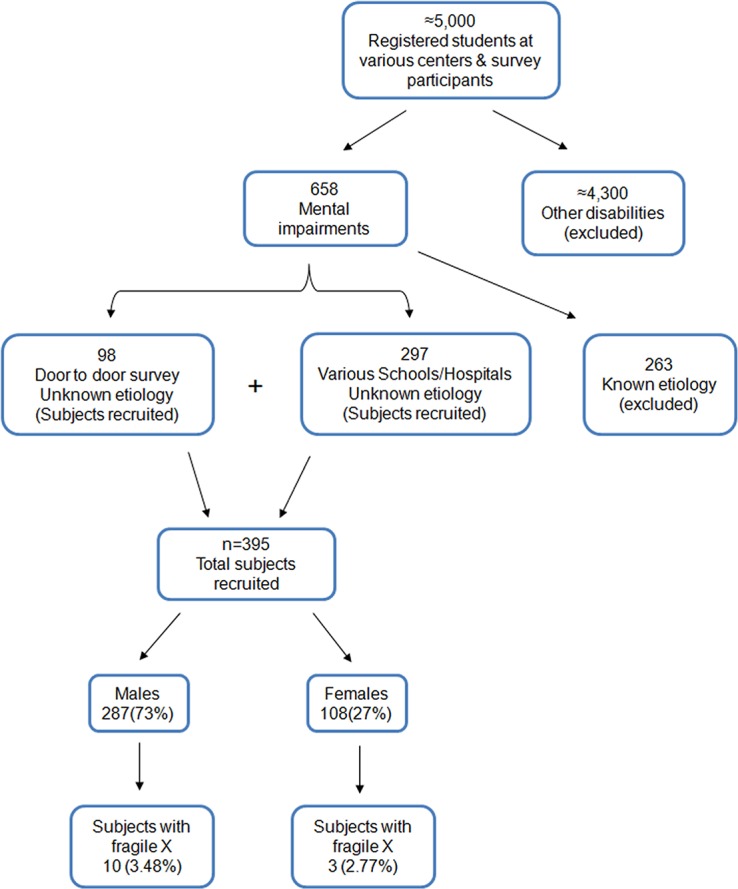
Survey plan for screening of subjects.

A cross sectional study was designed for recruitment of subjects with ID of unknown origin. Sampling of individuals was carried out during the years 2008–2012 at schools of special education/need, hospitals and through door to door surveys (conducted by MK, SI, MA and KM) from various regions of Pakistan including Central, Northern and Southern Punjab, Khyber Pakhtun Khwa (KPK) and the Islamabad capital territory. Most of the subjects (57%) belonged to Punjabi ethnic group, followed by Pathan (18%) where as others belonged to Kashmiri, Sindhi, Parsi, Balochi and Mohanna ethnic groups. These ethnic groups were determined with reference to their place of origin, language spoken and their religion. The study was conducted according to the ethical guide lines of the Helsinki-II Declaration and formal approvals from the ethical review committees of the Institute of Biomedical and Genetic Engineering, Islamabad and Quaid-i-Azam University, Islamabad were obtained. Samples of blood were drawn after written informed consents from parents, guardians, and/or institutional heads on behalf of the recruited subjects as outlined in PLOS consent form, for use in research and for publication of relevant case details. Minimum sample size required for determining the prevalence was estimated by analyzing previous reports [21][9] and also by using the formula n = (Z_(1-α)_^2^ (P(1-P)/D^2^) where, Z is for normal distribution at 5% α; P is estimated prevalence (3.0%); D is type II error. From an approximately 5,000 disabled individuals screened, 395 subjects with ID fulfilled the inclusion criteria and were subsequently recruited and sampled (Fig 1). Out of these 12, 179 and 120 individuals were from Central, Northern and Southern Punjab respectively; 53 from North West KPK and 31 from Islamabad capital territory. Each recruited subject underwent thorough medical examination by a medical practitioner. Subsequently, detailed clinical history, behavioral, psychological and physical characteristics were obtained. Individuals with known etiology of ID, i.e. Down syndrome, cerebral palsy, microcephaly or the ones due to traumatic cases were excluded. Children/subjects whose parents/guardians declined to share their information were also excluded. Recruited subjects with a family history of ID were further investigated and detailed pedigrees were constructed.

### Physical and behavioral features

Physical and psychiatric/behavioral attributes were recorded on performas as ‘absent’, ‘present’ or ‘borderline/present in the past’. Physical features included hyper extensible MP joints, facial features included long face, large or prominent ears, prominent jaw and prominent forehead. Subjects with at least two or more characteristic facial features were classified as ‘positive’ for typical FXS features. Psychiatric/behavioral features included attention deficit, social deficit speech disorder, poor eye contact, hyperactivity, tactile defensiveness, hand flapping and hand biting.

### Conventional PCR amplification of *FMR1* gene

DNA was extracted from peripheral blood mononuclear cells by using standard phenol-chloroform method. Control samples for normal, premutation carrier and individuals with a full mutation were obtained from the MIND Institute, UC Davis, California.

CGG repeat region within the *FMR1* was amplified by PCR. Two sets of repeat specific primer pairs (pairs 1 & 2; S1 Table) were employed [14,22]. The amplification was carried out in a 25μl reaction volume containing 75mM Tris-HCl (pH8.8), 1.5 mM MgCl_2,_ 20.0 mM (NH_4_)_2_SO_4_, 250 μM dNTPs, 1.25μM each of forward and reverse primers (Pair 1 or 2), 1.0 units of *Taq* polymerase (Fermentas, Germany) and 100ng DNA. The PCR was stabilized for GC rich region by using a stabilizer composed of 5M betaine, 5μl of 0.5M BSA, 10% DMSO, 6.7μl of 0.1M DTT and 27.6μl of dH_2_O for a total volume of 100μl. DMSO and PCR stabilizer were added 2μl each, accordingly. Initial denaturation of the samples was carried out at 95°C for 5 min, followed by 35 cycles each consisting of 95°C for 1 min, 62°C for 1 min, 72°C for 1 min. with a final extension at 72°C for 10 min. PCR products were analyzed by electrophoresis on 2% Ethidium Bromide stained agarose gels at 120V for 30 min. and 12% polyacrylamide gels at 20 Watts overnight.

### Bisulfite modification of DNA and methylation specific (modified) PCR

DNA was modified by bisulfite modification kit (Invitrogen, USA) before its use in methylation specific PCR (MS-PCR). Two primer sets were employed for this purpose (pairs 3 and 4, S1 Table). PCR amplification conditions were adopted from Panagopoulos *et al*., 1999 and were accordingly modified as follows: for methylation specific PCR, a 25μl reaction was performed containing 1xbuffer, 2mM MgCl_2,_ 0.2mM dNTPs, 0.5μM of each primer of the set 3 and 4 and 1.0 u of *Taq* polymerase. Bisulfite treated DNA (2.0 μl) was added to the reaction mixture and a total volume of 25μl were adjusted with dH_2_O[23]. Thermal cyclic conditions (for pair3) were as follow: initial denaturation at 94°C for 5 min, followed by 32 cycles at 93°C for 30 sec, 65°C for 30 sec. and 72°C for 30 sec., and final extension at 72°C for 10 min. Thermal cyclic conditions for primer pair 4 were: denaturation at 94°C for 5 min., followed by 32 cycles at 93°C for 1 min, 58°C for 1 min., and 74°C for 2 min., and a final extension at 74°C for 10 min. PCR products were separated by electrophoresis on 2% Ethidium Bromide stained agarose and 12% polyacrylamide gels at 120 V for 40 min. A 100 bp ladder was used as size standard.

### Southern Blotting

Southern blot analysis was performed to resolve full mutation and premutation alleles. Total genomic DNA (7–10μg) was digested using *Eco*RI and *Nru*I restriction enzymes that cleaved the unmethylated CpG sites in *FMR1*. Full mutation and premutation alleles and their repeat numbers in males as well as females were identified as previously described [19,22]. Positive controls were included in each analysis.

## Results

**Fig 2:**
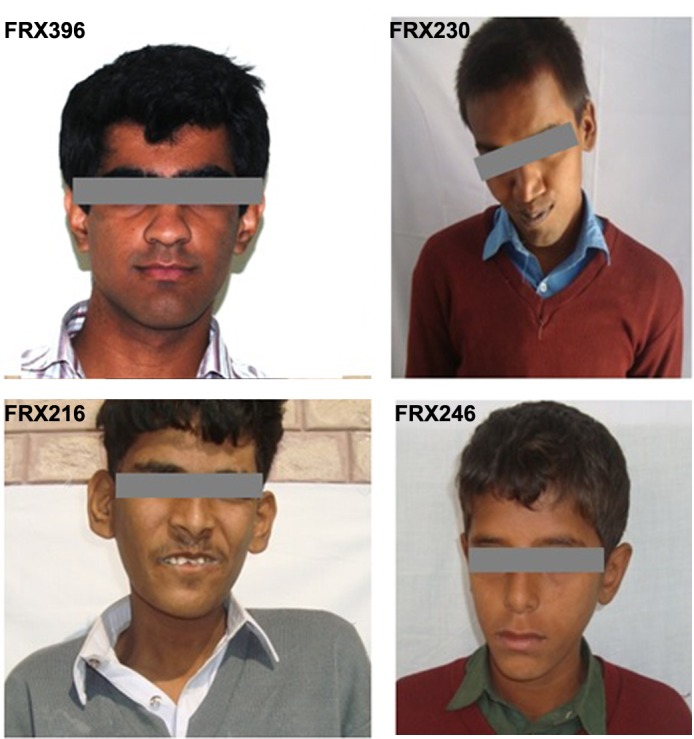
Facial features of unrelated Fragile X patients.

**Fig 3:**
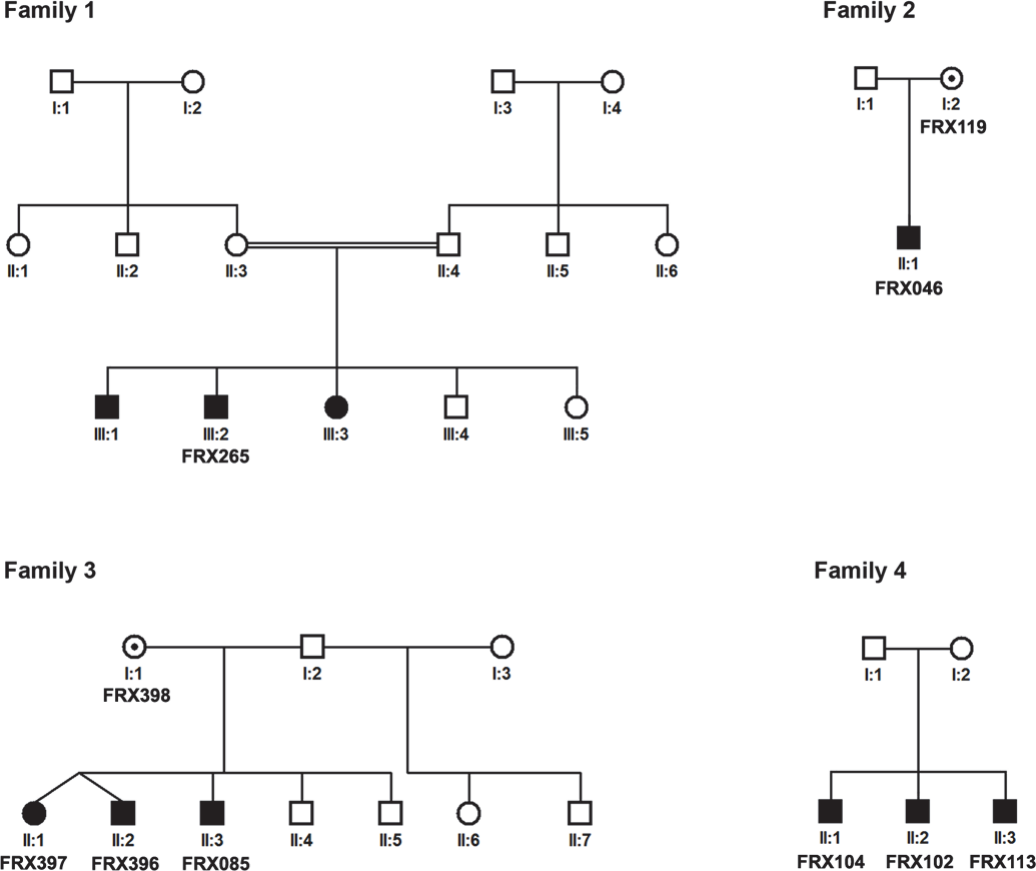
Selected pedigrees of FXS patients. Analyzed patients and carrier mothers are given identification numbers.

Data recorded for the 395 recruited subjects is summarized in Table 1. From our 395 subjects (age 14.28±7.01, range 4–40 years), 287 (73%) were males and 108 (27%) were females. All recruited individuals had ‘mild’ to ‘moderate’ ID, majority of them were hyperactive (96%), a number of them were tactile defensive (82%) and had speech disorder (79.0%). Quite a number among them showed hand biting (58%)and hand flapping (51%), poor eye contact was also observed (39.6%), while only 40 (10%) depicted all characteristic facial features of FXS, i.e., long narrow face, protruding forehead, large and prominent ears and prominent jaw. Moreover only 12% (n = 47) showed no characteristic facial features at all. A total of 26 (8.0%) subjects also had siblings with ID. Subjects diagnosed with FXS were 13 out of 395, most of them did not have typical FXS features (Fig 2) although some had sibling with ID (Fig 3). The subjects showing a family history of ID were investigated further and offered clinical genetic counseling. It was observed that unaffected males in the family shared no phenotypic resemblance to their affected male relatives. Affected siblings were able to cope well with daily-life skills especially those from special education centers. Obligate-carrier females had no remarkable physical findings.

**Table 1:** Clinical features of subjects with ID of unknown origin (n = 395, confirmed FXS = 13).

Symptoms Reported	Symptoms Not Present n(%)	Symptoms Present n(%)	Confirmed FXS Subjects n(%)
Hyperactivity	15 (*4*)	329 (*96*)	9(*69*)
Tactilely defensive	56 (*17*)	266 (*83*)	5 (*38*)
Hand flapping	162 (*49*)	167 (*50*)	5 (*38*)
Hand biting	140 (*42*)	192 (*58*)	4 (*3*1)
Poor eye contact	205 (*60*)	134 (*40*)	8 (*62*)
Speech disorder	64 (*20*)	251 (*80*)	8 (*62*)
Hyper extensible MP* joints	158(*72*)	59 (*27*)	2 (*15*)
Facial features	47 (*12*)	339 (*88*)	6 (*46*)
- Long face	226 (*66*)	113 (*34*)	5 (*38*)
- Prominent forehead	298 (*88*)	41 (*12*)	3 (*23*)
- Prominent jaw	278 (*82*)	61 (*18*)	6 (*46*)
- Prominent/long ears	215 (*63*)	124 (*37*)	6 (*46*)
Families with more than one affected individual	298 (*92*)	26 (*8*)	6 (*46*)

* metacarpophalangeal.

**Fig 4:**
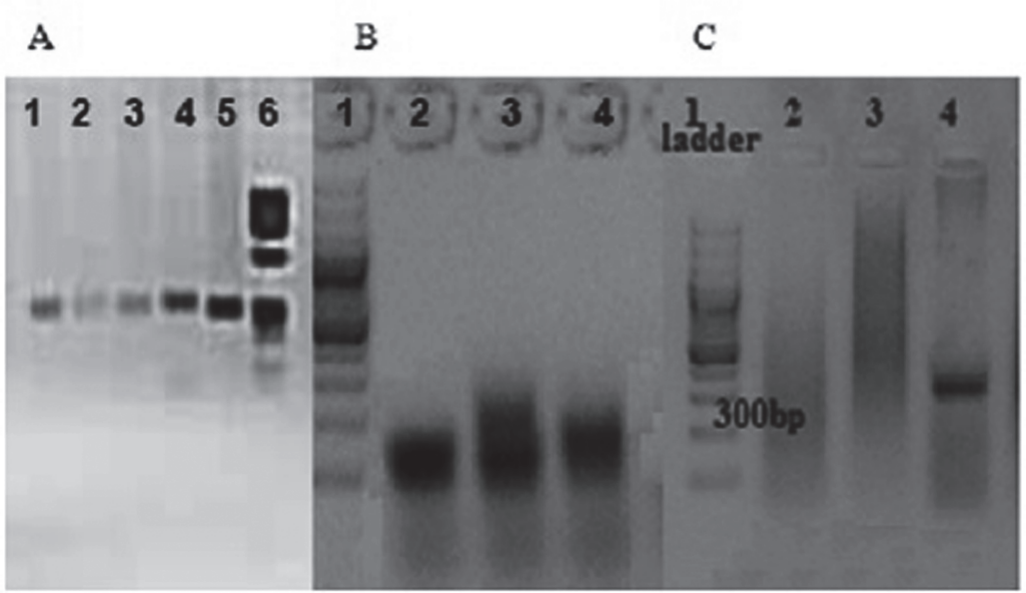
A. Primer pair 1 PCR amplification products of FXS negative subjects (lanes 1–5). Agarose gel electrophoresis results of methylation specific PCR. **B.** Primer pair 3 gave amplification product of ~80bp of FXS positive subjects (lanes 2–4). **C.** Primer pair 4 did not give amplification of the FXS positive subjects (lanes 2&3) but yielded a product of ~300bp with FXS negative subjects (lane 4).

**Fig 5:**
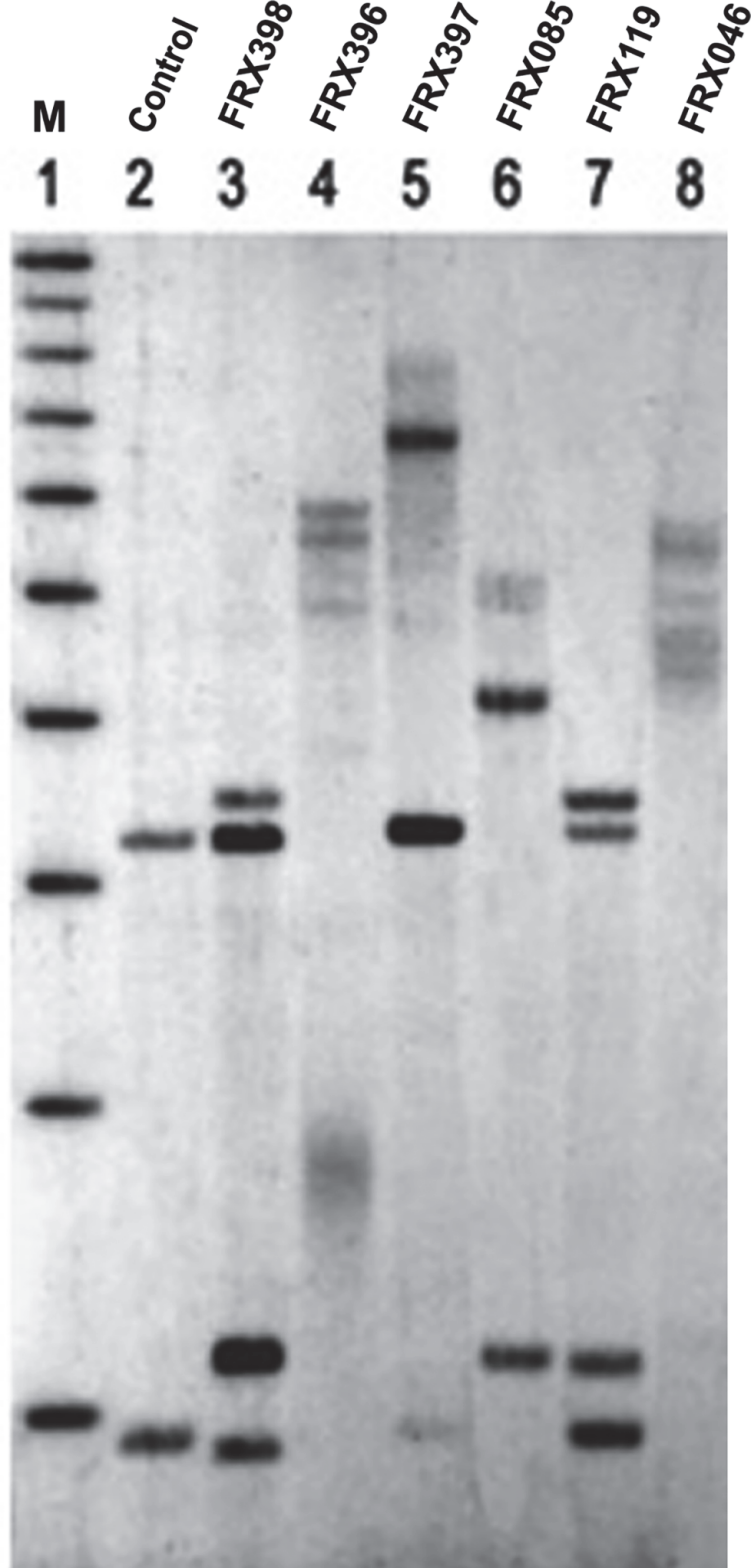
Southern blot analysis shows a normal female control (lane 2); premutation carrier females (lanes 3 and 7); methylation mosaic male (lane 4); full mutation female (lane 5); size mosaic males (lanes 6 and 8). 1kb DNA size marker is shown in lane 1.

In the conventional PCR reaction 13 individuals did not give any amplification product despite repeat trials while fragment sizes of 267 and 277 bp with primer sets 1 and 2, respectively were observed for rest of the samples (Fig 4A). Subsequent analyses using MS-PCR after bisulfite modification of DNA using two primer sets (Pairs 3 and 4) confirmed the male patients (Fig 4B and 4C) [23]. Southern blot analysis was performed on all FXS suspected subjects and their mothers. Obtained results verified the FXS males and the status of carrier females (Fig 5).

Hence out of 395 ID identified subjects, 13 (10 males and 3 females) were suspected for FXS in the initial screening through conventional PCR suggesting a prevalence of 3.5% and 2.8% among the male and female subjects respectively (mean 3.3%; proportion 0.033; 95%CI: 0.0153–0.0505). FXS was subsequently confirmed by MS-PCR as well as by Southern blotting.

## Discussion

Diagnosis of fragile X syndrome is complex when based on clinical evidence alone. The gender of carrier parents, gender of children and the size of CGG repeats are crucial parameters that influence disease expression. The broad phenotype can include intellectual disability, Attention Deficit Hyperactivity (ADHD), behavioral problems, autism spectrum disorders (ASD) and seizure [24][6]. The complex pattern of inheritance poses a great challenge for accurate diagnosis of the disease and genetic counseling [25]. We mainly scored the four facial features and some behavioral characteristics associated with FXS in order to evaluate their specificity with fragile X syndrome and found no specific clinical features for FXS determination. All the studied features were more prevalent in general ID population.

Pakistan is a country with high rate of consanguineous marriages due to a close structured society. Families have large sib-ships and extended generations. According to the last census (Population Census Organization, 1998), it was estimated that ±8.0% of the total Pakistani population has intellectual disabilities. Province-wise distribution showed that Punjab had 8%, KPK 7%, Sindh 7%, Baluchistan 6%, and Islamabad had 8% individuals with certain types of intellectual disabilities. It was therefore assumed that a substantial number of individuals with ID of unknown etiology might be patients of FXS due to a concentrated gene pool of defective alleles in the population. Hence, the primary goal of this study was to develop and establish molecular diagnostic tool for FXS in Pakistan and to estimate its prevalence in the region. Since karyotyping and ELISA based methods are nonspecific and less sensitive, PCR-based methods including MS-PCR were primarily employed. In the studied pool, samples of patients that did not amplify in conventional PCR were suspected as FXS patients, which was confirmed by MS-PCR and Southern blot analysis. Similar to the conventional PCR, MS-PCR was optimized by changing thermal cyclic conditions so that the sets of two primer pairs enabled to rule out false positive results. One of the limitations of the MS-PCR is that it cannot be reliably used for females as the method is unable to distinguish between female subjects who are carrier of premutation, full mutation and normal, all of whom show the same allelic band pattern following electrophoresis and also the allelic status of expanded repeat cannot be ascertained. In addition, MS-PCR has the disadvantage of degradation of DNA during modification procedure. FXS females could only be confirmed by Southern blotting which remains a labor intensive method. However with local modifications, protocols of conventional and MS-PCR developed by Fu *et al*. (1991), and Panagopolous*et al*., (1999) [23], respectively, have proved to be quite useful in diagnosing asymptomatic FXS subjects in many other populations like Chinese [21], Indian [26], Thai [27] and Korean [28].

Using this approach we identified 13 individuals with FXS which resulted in an overall prevalence of 33/1,000 among the recruited ID population, a figure similar to the worldwide prevalence [29]. These patients were individually evaluated for the FXS symptoms. It was found that hyperactivity, after intellectual disabilities, was one of the most common signs (present in 96%), while in few FXS patients hyperactivity was absent (4%). Characteristic FXS facial features were not always noticeable, some affected members of the family with FXS had only prominent jaw and protruding forehead. Behavioral changes were less obvious in the diagnosed subjects and varied considerably from individual to individual. No social isolation was observed and most of the affected subjects were friendly but docile with only a few exceptions.

The high figure of ID population is alarming for a developing country like Pakistan. Successful implementation of rehabilitation and training programs for ID subjects are highly dependent on correct diagnosis based upon etiology, manifestation and severity. Hence, it is vital to adopt a highly subjective training and education plan for ID individuals of a respective diagnostic category. Using appropriate diagnostic methodology it is possible to reduce this burden by genetic counseling of families and implementation of prenatal testing. More importantly it is recommended to carry out planned carrier screening. It is also possible to substantially improve the behavioral and social status of ID individuals and to make them participate in society.

In general clinical practice in Pakistan, the patients are diagnosed with ID in the ‘mild’, ‘moderate’, ‘severe’ or ‘profound’ categories while no further diagnostics are performed. Thus, towards this end, the current study represents a positive contribution for the molecular diagnosis of FXS in Pakistan. Medical practitioners can perform the simple conventional PCR for FXS diagnosis.

Currently, no cure is available for subjects affected with FXS. Therapeutic interventions in special education or day care are the only way to help individuals with FXS to be able to participate in society. In addition, support from other family members and sibs, community activists and non-governmental organizations is required for better management of patients of inherited ID in a developing society. We anticipate that a better diagnosis, customized focused rehabilitation and training programs would help in improving the status of individuals with intellectual ailments.

## Supporting Information

S1 Table Sequences and properties of oligos employed in conventional and methylation specific PCR of *FMR1*. (DOCX)

S2 Table Detailed clinical features of confirmed FXS patients. (XLSX)
