# Supplementary material for: Molecular Diagnosis of Fragile X Syndrome in Subjects with Intellectual Disability of Unknown Origin: Implications of Its Prevalence in Regional Pakistan
Source: PLoS One. 2015 Apr 14;10(4):e0122213. doi: 10.1371/journal.pone.0122213 (PMC4396850; doi:10.1371/journal.pone.0122213)
Supplement: S1 Table — (DOCX) [file pone.0122213.s001.docx]

**Table S1. Sequences and properties of oligos employed in conventional and methylation specific PCR of *FMR1.***

| **Pair** | **Sequence (5’→3’)** | **Annealing Temp. ºC** | **Allele size (bp) in normal subjects** | **Allele size (bp) in FXS suspects** | **Exon** | **Genomic coordinate*** |  |
| --- | --- | --- | --- | --- | --- | --- | --- |
| ***conventional PCR^1^*** | | | | | | | |
| 1 | GCTCAGCTCCGTTTCGGTTTCACTTCCGGT AGCCCCGCACTTCCACCACCAGCTCCTCCA | 58 | 281 | >300 | I | 146,993,448-146,993,728 |  |
| 2 | GCTCCGTTTCGGTTTCACTTCCGGTGGAGG  CGCACTTCCACCACCAGCTCCTCCATCTTC | 54 | 270 | >300 | I | 146,993,453-146,993,723 |  |
| ***methylation specific PCR^2^*** | | | | | | | |
| 3 | CGTCGTCGCGTTGTCGTAC  AACGACGAACCGACGACG | 65 | No amplification | ~75 | I |  |  |
| 4 | GGGAGTTTGTTTTTGAGAGGTGGG  CAACCTCAATCAAACACTCAACTCCA | 58 | ~300 | No amplification | I |  |  |

* UCSC Genome Browser: <http://genome.ucsc.edu/index.html?org=Human&db=hg19&hgsid=268739887> (accessed: Jan. 2012)

1. Fu YH, Kuhl DP, Pizzuti A, Pieretti M, Sutcliffe JS, Richards S, Verkerk AJ, Holden JJ, Fenwick RG, Warren ST. 1991. Variation of the CGG repeat at the Fragile X site results in genetic instability: resolution of the Sherman paradox. *Cell* 67: 1047-1058.

2. Panagopoulous I, Lassen C, Kristoffersson U, Aman P. 1999. A methylation specific approach for detection of Fragile X syndrome. *Hum Mut* 14: 71-79.
